# Fluoxetine elevates allopregnanolone in female rat brain but inhibits a steroid microsomal dehydrogenase rather than activating an aldo-keto reductase

**DOI:** 10.1111/bph.12891

**Published:** 2014-11-24

**Authors:** J P Fry, K Y Li, A J Devall, S Cockcroft, J W Honour, T A Lovick

**Affiliations:** 1 Department of Neuroscience, Physiology and Pharmacology, University College London (UCL)London, UK; 2 Institute of Women's Health, University College London (UCL)London, UK; 3 Department of Chemical Pathology, University College London HospitalLondon, UK; 4 School of Clinical and Experimental Medicine, University of BirminghamBirmingham, UK; 5 School of Physiology and Pharmacology, University of BristolBristol, UK

## Abstract

**Background and Purpose:**

Fluoxetine, a selective serotonin reuptake inhibitor, elevates brain concentrations of the neuroactive progesterone metabolite allopregnanolone, an effect suggested to underlie its use in the treatment of premenstrual dysphoria. One report showed fluoxetine to activate the aldo-keto reductase (AKR) component of 3α-hydroxysteroid dehydrogenase (3α-HSD), which catalyses production of allopregnanolone from 5α-dihydroprogesterone. However, this action was not observed by others. The present study sought to clarify the site of action for fluoxetine in elevating brain allopregnanolone.

**Experimental Approach:**

Adult male rats and female rats in dioestrus were treated with fluoxetine and their brains assayed for allopregnanolone and its precursors, progesterone and 5α-dihydroprogesterone. Subcellular fractions of rat brain were also used to investigate the actions of fluoxetine on 3α-HSD activity in both the reductive direction, producing allopregnanolone from 5α-dihydroprogesterone, and the reverse oxidative direction. Fluoxetine was also tested on these recombinant enzyme activities expressed in HEK cells.

**Key Results:**

Short-term treatment with fluoxetine increased brain allopregnanolone concentrations in female, but not male, rats. Enzyme assays on native rat brain fractions and on activities expressed in HEK cells showed fluoxetine did not affect the AKR producing allopregnanolone from 5α-dihydroprogesterone but did inhibit the microsomal dehydrogenase oxidizing allopregnanolone to 5α-dihydroprogesterone.

**Conclusions and Implications:**

Fluoxetine elevated allopregnanolone in female rat brain by inhibiting its oxidation to 5α-dihydroprogesterone by a microsomal dehydrogenase. This is a novel site of action for fluoxetine, with implications for the development of new agents and/or dosing regimens to raise brain allopregnanolone.

**Table:** Tables of Links

TARGETS	
**Enzymes***^a^*2013a
5α-reductase
Retinol dehydrogenase
**Transporters***^b^*2013a
5-HT transporter (SLC6A4)
**Ligand-gated ion channels**^*c*^2013a
GABA_A_ receptors

**Table:** 

LIGANDS
Allopregnanolone
Fluoxetine
Imipramine
Norfluoxetine
Progesterone

These Tables list key protein targets and ligands in this article which are hyperlinked to corresponding entries in http://www.guidetopharmacology.org, the common portal for data from the IUPHAR/BPS Guide to PHARMACOLOGY (Pawson *et al*., 2014) and are permanently archived in the Concise Guide to PHARMACOLOGY 2013/14 (^*a,b,c*^Alexander *et al*., 2013a,b,c[Bibr b1],[Bibr b2],[Bibr b3]).

## Introduction

Already widely prescribed as antidepressants, selective serotonin reuptake inhibitors (SSRIs), such as fluoxetine, have gained increasing acceptance since the 1990s (Steiner *et al*., 1995) as treatments for premenstrual dysphoric disorder (PMDD) and the more common but loosely defined premenstrual syndrome (see Marjoribanks *et al*., 2013). The former is a debilitating condition (Dennerstein *et al*., 2010) that includes both mood and physical symptoms such as irritability, anxiety, outbursts of anger, fatigue, sleep disturbance and hyperalgesia, and is thought at least in part to be triggered by the withdrawal of endogenous progesterone (see Backstrom *et al*., 2003). One explanation for the utility of fluoxetine and other SSRIs in the treatment of PMDD is their ability to raise concentrations of the neuroactive progesterone metabolite allopregnanolone in the CNS, an effect documented in the brains of male rats (Uzunov *et al*., 1996) and mice (Pinna *et al*., 2003) and in both male and female human CSF (Uzunova *et al*., 1996). Studies in male mice have shown the acute, fluoxetine-induced increase in brain allopregnanolone to occur at doses lower than those required for inhibition of 5-HT uptake, providing a possible explanation for the rapid response of PMDD patients to low doses of this drug (see Pinna *et al*., 2006 and Discussion). Indeed, there is increasing evidence that the effectiveness of SSRIs and certain other drugs in the treatment of a range of anxiety and depressive disorders may be explained, at least in part, by their ability to raise brain allopregnanolone concentrations (see Schule *et al*. 2014).

The anxiolytic, anticonvulsant and sedative steroid allopregnanolone is a potent enhancer of the actions of the inhibitory transmitter GABA at GABA_A_ receptors (see Wang, 2011). Moreover, withdrawal of progesterone in rats, either naturally as occurs during the ovarian cycle or by termination of pseudopregnancy or experimentally, after treatment with exogenous steroid, leads to changes in GABA_A_ receptor subunit expression which predispose to anxiety, seizures and hyperalgesia (Smith *et al*., 1998a,b[Bibr b48],[Bibr b49]; Griffiths and Lovick, 2005). These changes are mediated not directly by progesterone but rather by its metabolite allopregnanolone (Smith *et al*., 1998a; Gulinello *et al*., 2001; Turkmen *et al*., 2011). Thus, studies in rat suggest PMDD is triggered by progesterone withdrawal and mediated by a fall in the concentration of its neuroactive metabolite allopregnanolone in the brain, providing a rationale for treatment with fluoxetine.

**Figure 1:**
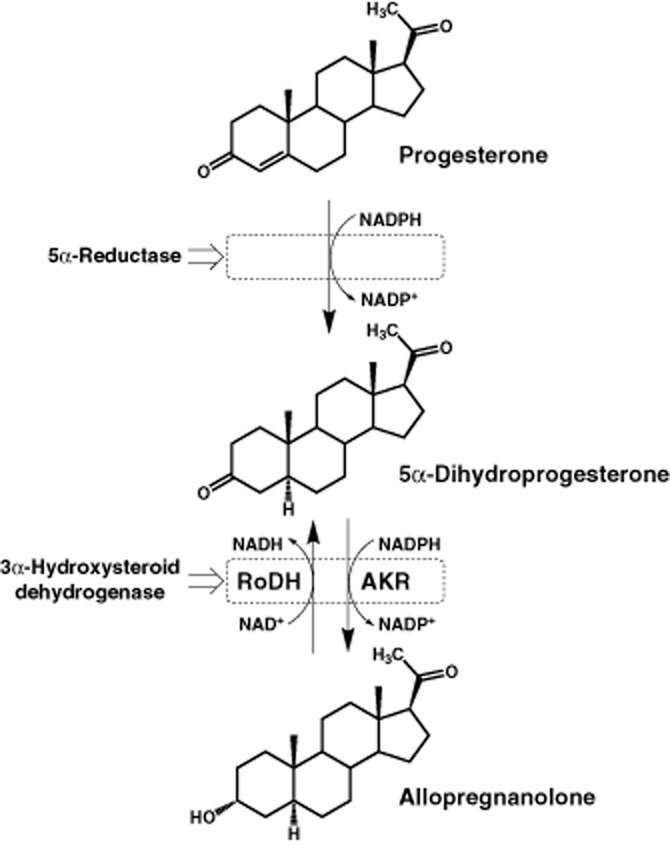
Synthesis of allopregnanolone from progesterone. A 5α-reductase enzyme produces 5α-dihydroprogesterone from progesterone utilizing NADPH as a co-factor. The activities of 3α-HSD can then either reduce 5α-dihydroprogesterone further to allopregnanolone or oxidize this allopregnanolone back to 5α-dihydroprogesterone. The reductive 3α-HSD *in vivo* is a cytosolic AKR utilizing NADPH as a co-factor whereas the oxidative 3α-HSD is a microsomal SDR, characterized as a RoDH and utilizing NAD^+^.

Synthesis of allopregnanolone from progesterone is catalysed by two enzyme systems (see Figure 1): a 5α-reductase producing 5α-dihydroprogesterone from progesterone and 3α-hydroxysteroid dehydrogenase (3α-HSD) activities interconverting 5α-dihydroprogesterone and allopregnanolone (see Compagnone and Mellon, 2000). In rat brain, the progesterone type 1 5α-reductase (SRD5A1) is an NADPH-dependent microsomal enzyme catalysing the irreversible reduction of progesterone to 5α-dihydroprogesterone. By contrast, 3α-HSD consists of two activities that are reversible *in vitro*: one cytosolic and NADP(H)-linked and the other NAD(H)-linked and found in particulate fractions. The cytosolic 3α-HSD activity is an aldo-keto reductase (AKR), with a higher affinity for NADPH than NADP^+^, ensuring that the enzyme functions predominantly as a ketosteroid reductase *in vivo* (see Penning, 2011). Only one gene has been identified for rat AKR, the type 1 3α-HSD liver enzyme AKR1C9 (Lin *et al*., 1999). The particulate NAD(H)-linked 3α-HSD activity is assigned to the short-chain dehydrogenase/reductase (SDR) family, which under normal cellular NAD^+^ : NADH ratios would be expected to function predominantly as oxidases (Miller and Auchus, 2011; Penning, 2011). Those responsible for oxidation of 3α-hydroxysteroids such as allopregnanolone have been identified as retinol dehydrogenase (RoDH) enzymes in human prostate (Bauman *et al*., 2006) and these microsomal RoDH enzymes are found also in rat (Napoli, 2001; Belyaeva and Kedishvili, 2006), where the predominant form in brain appears to be RoDH2 (Chai *et al*., 1995). Of the above enzymes, the 5α-reductase has the lowest *K*_m_ and is accepted as the first and rate-limiting step in the synthesis of allopregnanolone from progesterone (see Karavolas and Hodges, 1990).

Depending on brain region, the fluoxetine-induced elevation of allopregnanolone concentration in adrenalectomized and castrated male rat brains, as first reported by Uzunov *et al*. (1996), was accompanied by a fall or no change in 5α-dihydroprogesterone and no change in progesterone, implying an effect of the drug on 3α-HSD activity. Further evidence for 3α-HSD as a site of action for fluoxetine was obtained from experiments that showed this drug to cause an increased accumulation of allopregnanolone in brain slices loaded with 5α-dihydroprogesterone *in vitro* (Uzunov *et al*., 1996). Subsequently, Griffin and Mellon (1999) described an activation (decrease in *K*_m_) of purified recombinant rat liver type 1 (AKR1C9) and human type 3 (AKR1C2) 3α-HSD enzymes by fluoxetine, with no effect on rat type 1 5α-reductase activity in transfected cells. In contrast, Trauger *et al*. (2002) found no effect of fluoxetine or other SSRIs on the purified recombinant human type 3 (AKR1C2) 3α-HSD enzyme. Moreover and as mentioned above, 5α-reductase is considered the rate-limiting step in the synthesis of allopregnanolone from progesterone in brain and so activation of AKR would not be expected to raise concentrations of this metabolite *in vivo*. In an attempt to resolve these discrepancies and identify the mechanism by which fluoxetine elevates brain allopregnanolone, we have first investigated the effects of this drug on the concentration of allopregnanolone and its precursors progesterone and 5α-dihydroprogesterone in male and female rat brain, using short-term treatment with a low dose of this drug. Female rats were treated with fluoxetine at dioestrus, the stage of the ovarian cycle when secretions of progesterone, 5α-dihydroprogesterone and allopregnanolone are in decline (Ichikawa *et al*., 1974; Holzbauer, 1975). Using the same concentration of fluoxetine as employed in the conflicting reports of Griffin and Mellon (1999) and Trauger *et al*. (2002), we then compared the effects of this drug on the native reducing and oxidizing 3α-HSD enzyme activities of male rat brain *in vitro*. We used male rat brains for assays of native enzyme activity to reduce errors that might be caused by fluctuations in 3α-HSD activity during the ovarian cycle (see Karavolas and Hodges, 1990). For comparison, the latter investigations of native 3α-HSD included norfluoxetine, the major metabolite of fluoxetine, and imipramine, a tricyclic antidepressant. Finally, the actions of fluoxetine, norfluoxetine and imipramine were also tested on AKR and SDR (RoDH) activities of 3α-HSD expressed in transfected cells. Our results indicate that SSRIs elevate allopregnanolone in brain by inhibiting its oxidation back to 5α-dihydroprogesterone.

## Methods

### Animals

All animal care and experimental procedures were undertaken in accordance with the UK Animals (Scientific Procedures) Act 1986 and with the approval of the Animal Ethics Committee at the University of Birmingham. Studies are reported in accordance with the ARRIVE guidelines for reporting experiments involving animals (Kilkenny *et al*., 2010; McGrath *et al*., 2010). A total of 27 animals were used in the work described here.

Wistar rats, (males and females, 200–250 g body weight, 10–12 weeks old; from Charles River, UK), were housed in groups of two or three at 21 ± 1°C and a 12 h light/dark cycle (lights on at 07:00 h) with free access to food and water. Daily vaginal smears were taken at approximately 09:15 h from the females to establish their oestrus cycle stage (Brack *et al*., 2006) and to check for at least two normal cycles before the start of experiments. A final smear was taken after rats had been killed for brain samples, in order to confirm the cycle stage.

### Treatment with fluoxetine

Fluoxetine hydrochloride (1.75 mg·kg^−1^ i.p.) or the saline vehicle were administered to rats at 16:30–17:00 h on the evening prior to collection of brain samples, when the female rats were in early dioestrus. An additional dose was given at 09:00–10:00 h on the following morning (late dioestrus for the females) and animals killed 1 h later by decapitation. The whole brains minus olfactory lobes were rapidly removed, stripped of meninges, frozen on dry ice and stored at –80°C until analysis.

### Preparation of subcellular brain fractions

Whole male rat brains were homogenized using a Dounce homogenizer in 10 vol of ice-cold 5 mM HEPES buffer (pH 7.4) containing 0.32 M sucrose, one cOmplete® EDTA-free protease inhibitor cocktail tablet per 250 mL plus phenylmethyl sulphonyl fluoride (0.1 mM). This homogenate was centrifuged at 1000× *g* for 10 min at 4°C to yield the supernatant fraction S1 and pellet fraction P1 (nuclear). The pellet was resuspended in 5 vol of ice-cold 5 mM HEPES buffer (pH 7.4) containing 0.32 M sucrose and centrifuged at 1000× *g* at 4°C for a further 10 min. The resulting supernatant was pooled with S1. The pooled supernatant fraction was then centrifuged at 8000× *g* for 10 min at 4°C to yield a supernatant fraction S2 and pellet fraction P2 (mitochondrial). The remaining supernatant was centrifuged at 110 000× *g* for 60 min at 4°C to produce a supernatant cytosolic fraction S3 and pellet fraction P3 (microsomal). Fractions were frozen in dry ice and stored at −80°C. The protein content of each fraction was determined using the method of Bradford (1976), with BSA as a standard.

### Expression of recombinant enzymes

Plasmids pcDNA3 AKR1C9 (Ratnam *et al*., 1999) and pcDNA3 RODH4 LacZ (Bauman *et al*., 2006) were a gift from Professor T Penning (University of Pennsylvania, USA) and used to transfect HEK293 (HEK) cells cultured in DMEM supplemented with 10% fetal calf serum (FCS; heat-inactivated), 2 mM L-glutamine, 0.5 IU·mL^−1^ penicillin and 50 μg·mL^−1^ streptomycin. For transfection, the AKR1C9 and RoDH4 constructs were complexed with 10 μM polyethylenimine in Opti-MEM medium (Ehrhardt *et al*., 2006) and after 5 h, this medium replaced with DMEM containing 1% FCS. Following further culture for 24 h, the cells were washed with PBS before harvesting by trypsinization, centrifugation and a further wash in ice-cold PBS. They were then resuspended in 500 μL of SET buffer (250 mM sucrose, 1 mM EDTA and 50 mM Tris-HCl, pH 7.4 containing one cOmplete EDTA-free protease inhibitor cocktail tablet per 50 mL) and sonicated (4 × 10 s) on ice. Nuclei and unbroken cells were removed by centrifugation (1000× *g*, 5 min, 4°C) and the lysate then centrifuged at 100 000× *g* for 60 min at 4°C to obtain a supernatant cytosolic fraction. To remove contaminating cytosolic proteins, the membranes were resuspended in 1.5 mL of SET buffer and centrifuged (100 000× *g* for 60min). The membranes were finally resuspended in 750 μL of SET buffer. As for native brain fractions, protein content was determined (Bradford, 1976) and fractions stored at −80°C.

### TLC

Samples were applied to aluminium backed, silica gel 60-coated TLC plates and developed in one of the following solvent systems: (A) cyclohexane : n-butyl acetate 1:2 (v v^−1^); (B) cyclohexane : ethyl acetate 3:2 (v v^−1^); or (C) chloroform : ether 10:3 (v v^−1^). When necessary, the separated ^3^H-labelled steroids were visualized by exposing TLC plates to Fujifilm BAS-TR2040S imaging plates (Raytek Scientific Ltd., Sheffield, UK), which were then scanned in a Typhoon 9410 variable mode phosphorimager (GE Healthcare, Amersham, UK). Unlabelled steroid reference standards (50 μg) were visualized by exposure to iodine vapour.

### Measurement of brain steroids

Free steroids were extracted from brain for identification and assay by gas capillary chromatography-electron impact mass spectrometry (GC-EIMS) as described by Ebner *et al*. (2006). Each brain was homogenized with a Polytron homogeniser in 5 vol of ice-cold 5 mM KH_2_PO_4_ buffer (pH 7) then dripped into 20 vol 3% v v^−1^ acetic acid in ethanol 96% (w v^−1^) with sonication. The extract was centrifuged (28 000× *g*, 30 min, 25°C) and the supernatant de-lipidated by partitioning three times against 10 vol iso-octane. After drying down under vacuum and redissolving in 2 vol of 60% ethanol, the extract was further cleaned by passage through a 60 mg Oasis hydrophilic-lipophilic balanced (HLB) cartridge. Extracts were then dried under vacuum and redissolved in 20% ethanol before loading onto a 60 mg oasis mixed-mode anion exchange (MAX) cartridge, in order to separate free steroids from conjugates. After washing the MAX cartridge with 20% ethanol, the free steroids could be eluted in 4 mL ethyl acetate, dried down under nitrogen and stored in 1 mL 96% ethanol at −20°C until analysis.

Before derivatization for GC-EIMS analysis, the brain steroids were dried down under nitrogen together with the internal standard 16-dehydropregnenolone (50 ng). Methoxyamine hydrochloride (MO-HCl; 200 μL; 2% w v^−1^ in pyridine) was then added and samples incubated at 60°C for 1 h. This was followed by 100 μL of trimethylsilyl imidazole and a further incubation for 4 h at 120°C. Pyridine was then removed under nitrogen and the residue redissolved in 1 mL cyclohexane and washed twice against 0.5 mL of water. Finally, the cyclohexane was evaporated under nitrogen and the MO-trimethylsilyl ether derivatives redissolved in 5 μL of the same solvent ready for injection onto the GC-EIMS. Calibration standards at 0.625–10 ng were also derivatized for all steroids and with each set of samples.

All analyses were carried out on a Shimadzu 17A GC fitted with a 30 m long Zebron wall-coated open tubular column (Phenomenex, Macclesfield, UK) and coupled to a quadruple 5050A MS (Shimadzu, Milton Keynes, UK). Samples were injected in splitless mode at 280°C and a pressure of 200 kPa for 2 min. Thereafter, the pressure was decreased to 34.2 kPa for 5 min, followed by a rise of 6.5 kPa·min^−1^ to 81 kPa. After 0.33 min at this pressure, the gradient was set at 1.6 kPa·min^−1^ to a final pressure of 111.7 kPa, which was held for 2 min. The oven temperature was at 70°C for 5 min and then rose at 30°C·min^−1^ to 220°C. After 0.33 min, the gradient was 5°C·min^−1^ to 315°C, which was held for 10 min. The interface temperature was constant at 315°C.

For maximum sensitivity, the MS was run in selective ion monitoring mode, with two diagnostic ions for each steroid as follows: allopregnanolone (388 and 298), 16-dehydropregnenolone (415 and 384), 5α-dihydroprogesterone (343 and 288), progesterone (372 and 341). Steroids were only considered identified if the appropriate ions eluted at the same retention time and with the same ion ratios as shown by the standard compound (see Ebner *et al*., 2006). Quantitation was achieved by integrating these ion peaks and those for the internal standard and then comparing this response ratio with that obtained for the calibration standards.

### Enzyme assays

All enzyme assays were performed in triplicate at 37°C and in 100 mM potassium phosphate buffer (pH 7.4) containing 0.05 w v^−1^ BSA and 20 000 DPM (0.4 nM) of ^3^H-substrate in a total volume of 250 μL. For saturation assays, the ^3^H-substrate was increased to 40 000 DPM and combined with different concentrations of the non-radioactive steroid over the range 0.05–1.0 μM. Preliminary experiments established initial rate conditions, linear with respect to protein content and incubation time. Assay mixtures were set-up on ice and the incubations initiated by the addition to a final concentration of 1 mM of the cofactor β-NADPH or NAD^+^ to reductive or oxidative assays respectively. Buffer blanks and boiled sample controls were included in each assay. After the incubation, samples were returned to the ice bath and extracted with 1 mL of isooctane : ethyl acetate (1:1 v v^−1^) containing 0.5 μg·mL^−1^ of non-radioactive product steroid. This upper solvent layer was then dried down under nitrogen and spotted onto TLC plates for separation of substrate and product steroids. Outer lanes were spotted with non-radioactive standards. Assays of the reduction of 5α-dihydroprogesterone to allopregnanolone employed solvent system A to develop the plates whereas assays of the oxidation of allopregnanolone to 5α-dihydroprogesterone employed solvent system C. Once developed, the outer lanes were cut off and stained to identify the location of the product steroid. The corresponding regions in the sample lanes could then be cut out and placed in scintillation vials containing Ecoscint H (National Diagnostics, Hessle, UK) for measurement of radioactivity.

### Data analysis and statistical procedures

Diagnostic ion peaks on GC-EIMS traces were displayed using Shimadzu class 5000 software for manual integration. Phosphor images of ^3^H-labelled TLC plates were assessed using ImageQuant software (GE Healthcare). For enzyme assays, saturation with increasing concentrations of substrate or inhibition by increasing concentrations of fluoxetine was estimated by non-linear curve fitting in GraphPad Prism, (GraphPad Software, Inc., La Jolla, CA, USA) with 95% confidence limits on the parameters. Student's unpaired *t*-test was used to evaluate the significance of differences in brain concentrations of steroids between vehicle and fluoxetine-treated rats and of enzyme inhibition at single drug concentrations.

### Materials

Unless stated otherwise, all drugs, chemicals and reagents were purchased from (Sigma-Aldrich, Gillingham, UK or from VWR, Lutterworth, UK) and were of analytical grade. Solvents for brain steroid extraction and analysis by GC-EIMS were redistilled before use. The Oasis HLB and MAX cartridges were from Waters Corporation (Milford, MA, USA) while the silica gel 60-coated TLC plates came from VWR. Silanized glassware was used throughout. Non-radioactive standard steroids were purchased from Sigma-Aldrich or Steraloids Inc. (Newport, RI, USA) and [1,2,6,7-^3^H(N)]-progesterone (3666.7 GBq·mmol^−1^) from PerkinElmer (Buckinghamshire, UK). The latter was converted enzymically to [^3^H]-5α-dihydroprogesterone and [^3^H]-allopregnanolone by incubation at 250 nM with 10 vol homogenates of whole rat brain in 50 mM potassium phosphate buffer (pH 7.4) for 2 h at 37°C. The cofactor NADPH and dithiothreitol were each added at a final concentration of 1 mM. Steroids were extracted from the incubation with an equal volume of ethyl acetate : isooctane (1:1 v v^−1^) then dried down and spotted onto TLC plates for separation in solvent system A. Marker lanes spotted with non-radioactive standards were stained to reveal the location of the desired ^3^H-steroids, which were then cut out and eluted with 2 mL ethanol followed by 2 mL ethyl acetate. These eluates were dried down and spotted again onto TLC plates for further purification: [^3^H]- 5α-dihydroprogesterone in solvent system B and [^3^H]- allopregnanolone in solvent system C. Each label gave a single peak at Rf values coinciding with the appropriate non-radioactive standard.

## Results

### Rat brain steroids following treatment with fluoxetine

**Figure 2:**
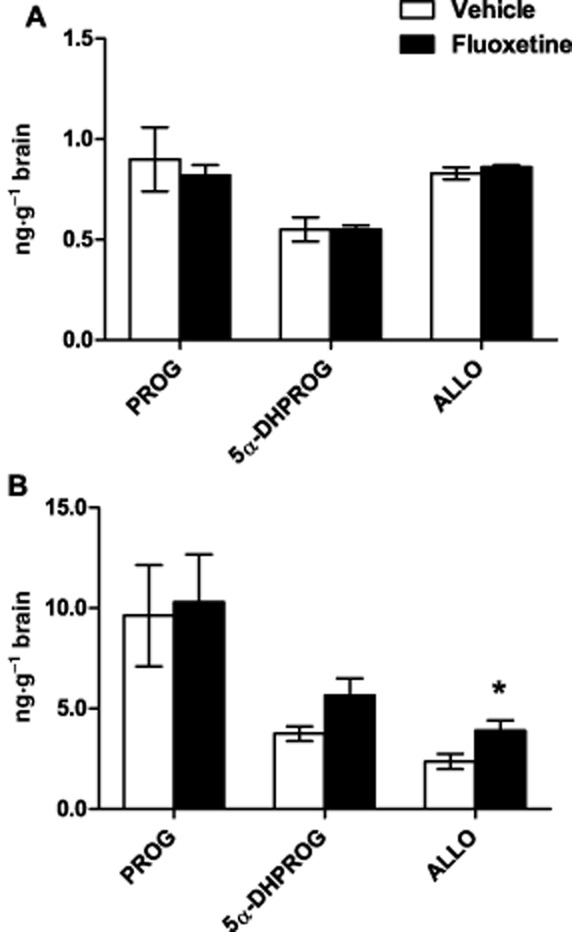
Effect of fluoxetine (FLX) treatment on the concentrations of progesterone (PROG), 5α-dihydroprogesterone (5α-DHPROG) and allopregnanolone (ALLO) in the brains of (A) male rats and of (B) female rats at late dioestrus (note difference in scale of *y*-axes). Two doses of fluoxetine hydrochloride (1.75 mg·kg^−1^ i.p.) were administered: one at 16:30–17:00 h on the day prior to collection of brain samples and another at 09:00–10:00 h on the following day 1 h before killing the animals. Vehicle-treated rats received an injection of the saline vehicle alone. All values mean ± SEM (*n* ≥ 4). *P < 0.05, significantly different to vehicle-treated control.

The steroids progesterone, 5α-dihydroprogesterone and allopregnanolone could be detected in the brains of both male rats (*n* = 4) and female rats (*n* = 6) at late dioestrus, although at around 10-fold lower concentrations in the males. Treatment of female rats in dioestrus with fluoxetine increased brain allopregnanolone concentrations (*P* = 0.035 in comparison with vehicle-treated controls) but no significant change in progesterone (*P* = 0.850) or 5α-dihydroprogesterone (*P* = 0.054). By contrast, when the same steroids were measured in the brains of male rats given the same treatment with fluoxetine, there were no significant changes in the concentrations of progesterone (*P* = 0.625), 5α-dihydroprogesterone (*P* = 0.960) or allopregnanolone (*P* = 0.416) (Figure 2).

### Actions of fluoxetine on progesterone metabolism in subcellular fractions of rat brain

**Figure 3:**
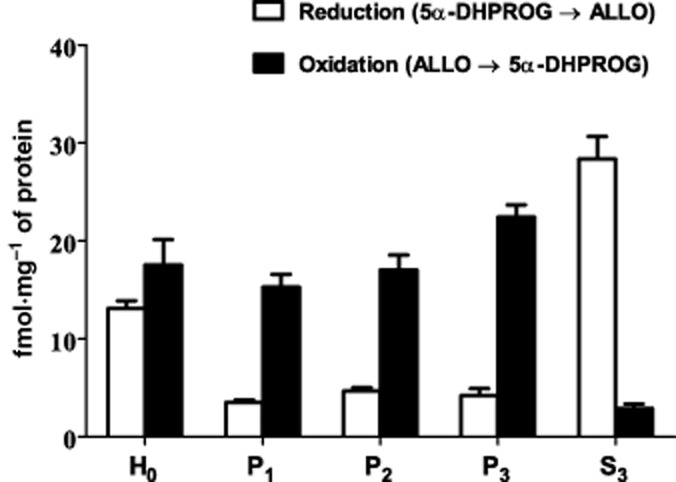
Activities of 3α-HSD in subcellular fractions of adult male rat brain: H_0_ whole homogenate, P_1_ nuclear, P_2_ mitochondrial, P_3_ microsomal and S_3_ cytosol. Reductive activity was measured by the conversion of 5α-dihydroprogesterone (5α-DHPROG) to allopregnanolone (ALLO) in the presence of NADPH and oxidative activity by the reverse reaction in the presence of NAD^+^. Activities are shown as mean ± SEM (*n* = 5) from assays in triplicate.

**Figure 4:**
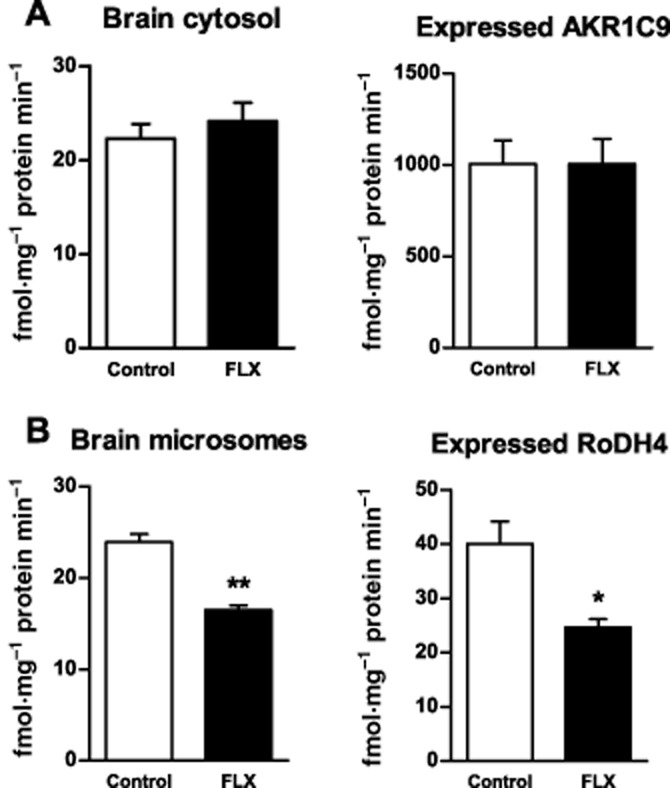
Effect of fluoxetine (FLX) at 50 μM on 3α-HSD activities: (A) the reducing activity producing allopregnanolone from 5α-dihydroprogesterone in male rat brain cytosol or expressed AKR1C9 and (B) the oxidizing activity producing 5α-dihydroprogesterone from allopregnanolone in male rat brain microsome fractions or expressed human RoDH4. Activities are shown as mean ± SEM (*n* ≥ 4) from assays in triplicate; *P < 0.01, **P < 0.001 compared with control.

**Figure 5:**
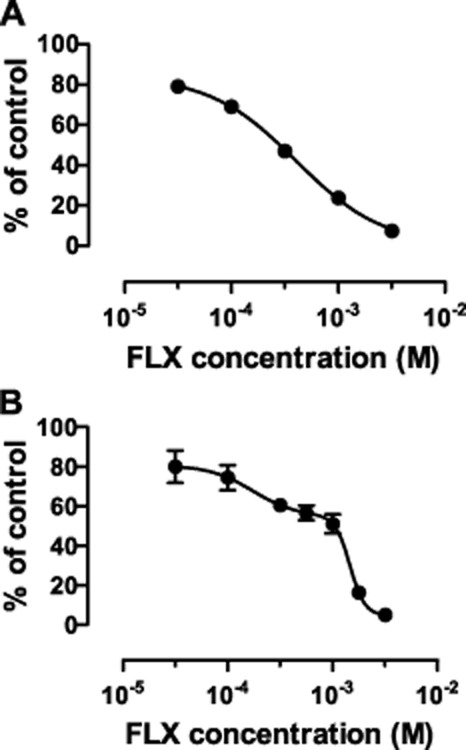
Effect of increasing concentrations of fluoxetine (FLX) on the oxidizing 3α-HSD activity producing 5α-dihydroprogesterone from allopregnanolone in (A) male rat brain microsomes (*n* = 4 brains) and (B) expressed human RoDH4 (*n* = 4 assays). Each assay was performed in triplicate and the overall means shown ± SEM (if not visible smaller than symbol for mean). Best fit curves are shown giving IC_50_ values (with 95% confidence limits in brackets) as follows: (A) 240 μM (216–268 μM) and (B) 174 μM (36–138 μM; fraction 0.34 of total activity) plus 1466 μM (1035–2070 μM).

Subcellular fractions of male rat brain showed the reducing activity of 3α-HSD, producing allopregnanolone from 5α-dihydroprogesterone, to be enriched in the cytosol whereas the oxidizing activity catalysing the reverse reaction was mostly particulate, with the highest activity in the microsomal fraction (Figure 3). When tested at 50 μM, fluoxetine had no effect on the reducing activity in the cytosol but inhibited the microsomal oxidation of allopregnanolone to 5α-dihydroprogesterone (Figure 4). Further assays of this microsomal oxidative activity over a range of fluoxetine concentrations gave an IC_50_ of 240 μM (Figure 5). When tested at this IC_50_ over a range of substrate allopregnanolone concentrations, fluoxetine was found to cause a non-competitive inhibition, with a significant decrease in *V*_max_ (control 51.48 ± 14.69 vs. fluoxetine 19.37 ± 3.58 nmol·mg^−1^ protein min^−1^) but not *K*_m_ (control 0.62 ± 0.34 vs. fluoxetine 0.44 ± 0.17 μM; all values ± 95% confidence limits). When tested at the IC_50_ of 240 μM for fluoxetine, a significant (*P* < 0.001) inhibition of microsomal 3α-HSD oxidative activity was also seen with norfluoxetine (50.32 ± 1.56%) and imipramine (43.74 ± 1.07%) and confirmed for fluoxetine (51.78 ± 1.99%) in comparison with controls (all values mean ± SEM % control).

### Actions of fluoxetine on AKR1C9 and RoDH4 activities expressed in HEK cells

When tested at 50 μM, fluoxetine had no effect on the reduction of 5α-dihydroprogesterone to allopregnanolone catalysed by the cytosol of HEK cells transfected with the plasmid for rat AKR1C9 (Figure 4A). In contrast and as found in the native microsomal fraction of rat brain, fluoxetine inhibited the oxidation of allopregnanolone to 5α-dihydroprogesterone catalysed by the membrane fraction of HEK cells transfected with the plasmid for human RoDH4 (Figure 4B). Assays of this RoDH4 activity over a range of fluoxetine concentrations gave a best fit to a biphasic inhibition, with estimated IC_50_ values of 140 and 1466 μM (Figure 5B). When tested at the IC_50_ value of 240 μM found previously for the effect of fluoxetine on native rat brain microsomes, a significant (*P* < 0.005) inhibition of RoDH4 oxidative activity was also seen with norfluoxetine (37.75 ± 0.95%) and imipramine (25.45 ± 0.04%) and confirmed for fluoxetine (55.43 ± 1.91%) in comparison with controls (all values mean ± SEM % control).

## Discussion and conclusions

The present study found the reducing and oxidizing activities of 3α-HSD to have distinct subcellular distributions in adult rat brain, in agreement with previous observations (Rommerts and Van der Molen, 1971; Krause and Karavolas, 1980). The activity catalysing reduction of 5α-dihydroprogesterone to allopregnanolone was predominantly cytosolic and presumably the AKR as purified by Penning *et al*. (1985). We found fluoxetine at 50 μM to have no effect either on this native enzyme or on the rat liver type I 3α-HSD AKR1C9 activity expressed in HEK cells. These results contradict the report of Griffin and Mellon (1999), who found this concentration of fluoxetine to activate (decrease the *K*_m_ of) the cloned and purified recombinant AKR1C9 protein catalysing the production of allopregnanolone from 5α-dihydroprogesterone and to have the same effect on the human AKR1C2 enzyme, an orthologue of the rat AKR1C9 (Lin *et al*., 1999). However, our results are consistent with the observations of Trauger *et al*. (2002), who found no effect of fluoxetine at 50 μM on the purified recombinant human AKR1C2 enzyme and suggested that the results of Griffin and Mellon (1999) might have been caused by polymers from the Prozac (Sigma-Aldrich) tablets used by the latter authors as a source of fluoxetine acting as molecular crowding agents and increasing the effective concentration of substrate in the assays.

In contrast to the lack of effect of fluoxetine on reductive 3α-HSD activity, the present study identified an inhibitory effect on the oxidative 3α-HSD activity, both in rat brain microsomal fractions and in the membranes of HEK cells transfected with human RoDH4. We were unable to obtain plasmid DNA for rat RoDH but human RoDH4 is the predominant form expressed in brain and shows 71% sequence identity to the rat RoDH2 (Belyaeva and Kedishvili, 2006), which is the predominant form expressed in the brain of this species (Chai *et al*., 1995) and displays 3α-HSD oxidative activity (Hardy *et al*., 2000). Moreover, protein sequences of human RoDH4 (also named RDH16; NCBI Reference: 075452.2) and rat RoDH2 (NCBI Reference: NP_954678.1) display 93 and 95% identical or related amino acid residues in the active site and the steroid binding site respectively. Inhibition of RoDH4 by fluoxetine in the present study gave a best fit to a biphasic curve suggesting multiple drug binding sites, as might be expected for this multimeric protein (see Liden and Eriksson, 2006; Penning, 2011). In contrast, inhibition of the oxidative 3α-HSD activity in rat brain microsomal fractions by fluoxetine gave a best fit to a single site. This difference between the native and expressed RoDH activities may be due to an averaging effect of isoenzymes in the former preparation, because at least one other RoDH has been detected in rat brain (Chai *et al*., 1995). There is also the possibility that RoDH4 requires a substrate binding protein for optimal activity, as appears to be the case for RoDHs (see Liden and Eriksson, 2006; Napoli, 2012) and which may be lacking in the HEK cells we used for expression of this enzyme. The inhibition of RoDH4 activity by fluoxetine revealed in the present study requires further investigation as to its mechanism of action. Nevertheless and in addition to the current relevance to the treatment of premenstrual dysphoria, the inhibition of RoDH4 reported here also suggests a possible use for fluoxetine in treating androgen-dependent diseases; by reducing the formation of 5α-dihydrotestosterone from 5α-androstan-3α,17β-diol (Bauman *et al*., 2006). However, inhibition of RoDH could also alter vitamin A metabolism, depressing the synthesis of all-*trans*-retinoic acid (Napoli, 2012) and may explain the decrease in bone mineral density seen on chronic treatment with fluoxetine (Rizzoli *et al*., 2012), especially in postmenopausal women (Studd, 2011).

Short-term treatment of female rats at dioestrus with fluoxetine produced changes in brain steroids consistent with the above observations *in vitro*. Thus, inhibition of allopregnanolone oxidation in the brain by fluoxetine treatment elevates the concentration of this steroid without a significant effect on 5α-dihydroprogesterone. Our results differ in the latter respect from those of Uzunov *et al*. (1996), who found the fluoxetine-induced elevation of allopregnanolone in whole male rat brain (but not all individual brain regions) to be accompanied by a fall in 5α-dihydroprogesterone concentration. Moreover, in contrast to the present results with females and to the previous results of Uzunov *et al*. (1996) with males, we failed to observe a fluoxetine-induced elevation of allopregnanolone in whole male rat brain. This discrepancy is unlikely to be due to procedural differences in the extraction and assay of brain steroids because the concentrations of allopregnanolone measured in whole brain of vehicle-treated male rats in the present study were comparable with those (0.8 ng·g^−1^) reported by Uzunov *et al*. (1996). However, Uzunov *et al*. (1996) used a dose of fluoxetine 10-fold higher than that employed in the present study. Taken together with the observations of Uzunov *et al*. (1996), therefore, our results suggest that female rats may be more sensitive to the brain allopregnanolone elevating action of fluoxetine than males. Clinical evidence also indicates that women are more sensitive to SSRIs such as fluoxetine, but not after the menopause (Kornstein *et al*., 2000; Khan *et al*., 2005; Pinto-Meza *et al*., 2006). The sex difference in responses to the low dose of fluoxetine reported here is unlikely to be due to differences in RoDH enzyme structure because male rat brain microsomal fractions were used to reveal the inhibitory effect of fluoxetine on the oxidation of allopregnanolone to 5α-dihydroprogesterone and a similar effect was observed on the overexpressed RoDH4 enzyme. More likely, the sex difference in response to fluoxetine reflects a disparity in the preponderant source of brain allopregnanolone. In male rat brain, where progesterone appears to derive predominantly from synthesis within this tissue rather than from plasma (Corpechot *et al*., 1993), the 5α-reductase enzyme would be the rate-limiting step in the production of allopregnanolone (see Introduction) and concentrations of this steroid expected to remain largely unaffected by inhibition of its oxidation back to 5α-dihydroprogesterone. Conversely, in female rat brain, the higher concentrations of progesterone, 5α-dihydroprogesterone and allopregnanolone derive predominantly from the ovaries via the plasma (Corpechot *et al*., 1993). If the supply of allopregnanolone from the plasma exceeded that produced within the brain from progesterone in our female rats, then inhibition of allopregnanolone oxidation by fluoxetine treatment would be expected to elevate the concentration of this steroid. As mentioned in the Introduction, we used male rat brain for studies of native 3α-HSD to avoid possible fluctuations in activity during the ovarian cycle. Clearly, further studies are now required to characterize the effects of fluoxetine on allopregnanolone metabolism in the female brain at different stages of this cycle.

When tested at the IC_50_ concentration of 240 μM for fluoxetine, both norfluoxetine and imipramine inhibited the oxidative 3α-HSD activity of rat brain microsomes and of expressed human RoDH4. Norfluoxetine is the major active metabolite of fluoxetine found in brain and has been reported to be more potent than the parent drug for elevation of mouse brain allopregnanolone (Pinna *et al*., 2003). However, our results for the tricyclic imipramine were surprising as systemic administration of this drug was reported to have no effect on rat or mouse brain allopregnanolone content (Uzunov *et al*., 1996; Pinna *et al*., 2003). Dosages of imipramine used in the latter studies are unlikely to have produced whole brain concentrations of this drug greater than 20 μM (Sugita *et al*., 1989), at least 10-fold lower than tested here against the oxidative 3α-HSD activity of the rat brain microsomal fraction and human RoDH4. Thus, our results with imipramine are probably not of therapeutic relevance but are nonetheless consistent with the report that 50 μM imipramine reduces the *V*_max_ for oxidation of allopregnanolone by recombinant rat type 1 3α-HSD *in vitro* (Griffin and Mellon, 1999). Moreover, the tetracyclic antidepressant mirtazapine, which elevates plasma allopregnanolone in humans, has also been shown to inhibit the overexpressed human microsomal 3α-HSD oxidizing allopregnanolone to 5α-dihydroprogesterone with an IC_50_ of 46 μM, yet have no effect at up to 50 μM on the recombinant human cytosolic type 3 (AKR1C2) 3α-HSD catalyzing the reduction of 5α-dihydroprogesterone to allopregnanolone (see Schule *et al*., 2006).

We selected a concentration of 50 μM for initial tests of fluoxetine against 3α-HSD activity because this was the concentration used in the conflicting reports of Griffin and Mellon (1999) and Trauger *et al*. (2002). It is also close to the steady-state concentration of 21 μM reached for fluoxetine in human brain during a typical 20 mg·day^−1^ p.o. adult dosing schedule (Henry *et al*., 2005). As for the dosage of fluoxetine given to the rats in the present study, this was chosen as the treatment we have shown previously to prevent the stress-induced hyperalgesia associated with the progesterone withdrawal of late dioestrus (AJ Devall *et al*., submitted) and would be expected to produce brain concentrations in the region of 10 μM for both fluoxetine and norfluoxetine (Qu *et al*., 2009). From studies of fluoxetine and norfluoxetine in mice, Pinna *et al*. (2004; 2003[Bibr b37],[Bibr b38]) conclude that these SSRIs elevate brain allopregnanolone content at doses below those necessary for inhibition of 5-HT reuptake *ex vivo*. A similar situation appears to hold in the rat, where doses as used in the present study have no effect on the amount of extracellular 5-HT collected by microdialysis in the dorsal periaqueductal grey *in vivo* (AJ Devall *et al*., submitted). Antidepressant actions of SSRIs mediated at least in part through 5-HT reuptake inhibition often require 3–8 weeks of treatment, during which down-regulation of 5-HT autoreceptors, adrenoreceptors and other changes are thought to occur (see Racagni and Popoli, 2008). By contrast, PMDD patients respond to fluoxetine within 2 days (Steinberg *et al*., 2012), as would be expected for a response mediated by the acute inhibition of RoDH enzymes documented in the present study.

To conclude, our results indicate that fluoxetine elevates the concentration of the neuroactive steroid allopregnanolone in the female rat brain not by enhancing its synthesis from 5α-dihydroprogesterone but by inhibiting its oxidation back to this inactive precursor by microsomal RoDH enzymes. As such, fluoxetine might be better described as a selective intracrine modulator (see Penning, 2011) than a selective brain steroidogenic stimulant (Pinna *et al*., 2006). Most importantly however, the present study identifies a new site of action for fluoxetine, with implications for the development of more selective agents and/or dosing regimens to raise brain allopregnanolone, thereby offering the potential to treat disorders of progesterone withdrawal such as PMDD and post-partum depression.

## Abbreviations

3α-HSD: 3α-hydroxysteroid dehydrogenase
AKR: aldo-keto reductase
GC-EIMS: gas capillary chromatography-electron impact mass spectrometry
HLB: hydrophilic-lipophilic-balanced
MAX: mixed-mode anion exchange
MO: methoxyamine
PMDD: premenstrual dysphoric disorder
RoDH: retinol dehydrogenase
SDR: short-chain dehydrogenase/reductase
SSRI: selective serotonin reuptake inhibitor
